# HIV-1 tropism prediction by the XGboost and HMM methods

**DOI:** 10.1038/s41598-019-46420-4

**Published:** 2019-07-10

**Authors:** Xiang Chen, Zhi-Xin Wang, Xian-Ming Pan

**Affiliations:** 1 Key Laboratory of Ministry of Education for Protein Science, School of Life Sciences, Tsinghua University, Beijing, 100084 China; 2 Key Laboratory of Ministry of Education for Bioinformatics, School of Life Sciences, Tsinghua University, Beijing, 100084 China

**Keywords:** HIV infections, Diagnosis

## Abstract

Human Immunodeficiency Virus 1 (HIV-1) co-receptor usage, called tropism, is associated with disease progression towards AIDS. Furthermore, the recently developed and developing drugs against co-receptors CCR5 or CXCR4 open a new thought for HIV-1 therapy. Thus, knowledge about tropism is critical for illness diagnosis and regimen prescription. To improve tropism prediction accuracy, we developed two novel methods, the extreme gradient boosting based XGBpred and the hidden Markov model based HMMpred. Both XGBpred and HMMpred achieved higher specificities (72.56% and 72.09%) than the state-of-the-art methods Geno2pheno (61.6%) and G2p_str (68.60%) in a 10-fold cross validation test at the same sensitivity of 93.73%. Moreover, XGBpred had more outstanding performances (with AUCs 0.9483, 0.9464) than HMMpred (0.8829, 0.8774) on the Hivcopred and Newdb (created in this work) datasets containing larger proportions of hard-to-predict dual tropic samples in the X4-using tropic samples. Therefore, we recommend the use of our novel method XGBpred to predict tropism. The two methods and datasets are available via http://spg.med.tsinghua.edu.cn:23334/XGBpred/. In addition, our models identified that positions 5, 11, 13, 18, 22, 24, and 25 were correlated with HIV-1 tropism.

## Introduction

Human Immunodeficiency Virus 1 (HIV-1) is a retrovirus which mainly infects T-lymphocytes, macrophages and dendritic cells^1^. HIV-1 enters into those host cells by chronologically interacting with primary receptors and co-receptors^2^. Fourteen co-receptors have been detected *in vitro*^3^. However, *in vivo*, the major co-receptors are CCR5 and CXCR4^4,5^. Indeed, a vast majority of subtype B and probably all subtype C HIV-1 positive individuals are initially infected via CCR5^2^. Viruses using CCR5 are known as R5 tropic, whereas viruses using CXCR4 are called X4 tropic. R5X4 or dual tropic viruses as a third class can bind to either CCR5 or CXCR4^6^. For simplicity, X4 and dual tropic viruses are called X4-using tropic.

R5 tropic viruses start the HIV-1 infection^7^. This start is shown by the HIV-1 resistance in individuals where the function of CCR5 is disabled by a homozygous *ccr5*-Δ32 gene^4,8,9^. Besides, X4-using tropic viruses are associated with disease progression, since those viruses emerge at the later stage of an infection in about half of the infected individuals^2,8,10–12^. Furthermore, Miraviroc (MVC), a CCR5 antagonist and the only FDA-approved entry inhibitor, binds to the hydrophobic transmembrane helices of CCR5 so as to allosterically inhibit viruses from entering^13^. It has been proved that MVC cannot transform R5 viruses into X4-using viruses^14,15^. Consequently, it becomes clear that tropism testing is necessary for several reasons: (1) To determine the illness progression^2,11^; (2) To decide whether MVC can be used^10^; and (3) To monitor changes in viral quasispecies in order to modify regimens in time^4^.

In the last decades, two kinds of tropism testing methods, phenotypic and genotypic, have been developed. The phenotypic methods, such as ES-Trofile, are expensive, time-consuming, poorly accessible due to requiring specialized centers, and cannot provide consistent results when the viral load is below 1000 copies/ml^16^. Thus, the application of these methods is limited in clinical routines in Europe^5,8^. Instead, the genotypic tropism testing is a preferred method due to low cost, reduced turnaround time and great accessibility, even when the viral load is below 1000 copies/ml^17^. In contrast to phenotypic methods, genotypic methods are based on statistics or machine learning. These methods analyze the third variable (V3) loop of the viral glycoprotein gp120, which predominantly determines its tropism^18^. The earliest proposed genotypic method for prediction of X4-using tropism is the 11/25 rule. This rule is based on the presence of a positively charged amino acid in positions 11 or 25 of the V3 sequence^19^. Other genotypic methods such as WebPSSM^20,21^ and CM^22^ predict tropism based on scores that are calculated from position specific score matrices (PSSMs). In detail, WebPSSM constructs ungapped PSSMs, while CM constructs gapped PSSMs and takes the 11/25 rule and net charge into consideration. Recently, many genotypic methods based on machine learning have also been published. The method Geno2pheno^4^ combines two machine learning approaches, support vector machine (SVM) and decision trees, and uses clinical information such as viral loads and CD4-cell counts if available. Another method from the same laboratory, G2p_str^23^, combines SVM and Lasso regression and uses the amino acid structure feature. Hivcopred^24^ is based on SVM^light^ with the split amino acid composition feature. T-CUP2^25^ employs random forests (RFs) with the structural information of hydrophobicity and electrostatic potential. Currently, Geno2pheno is the most widely used method and the only genotypic method recommended for usage in clinical routines by the European Consensus Group^5,8^.

Genotypic methods can predict R5 viruses (~90%) accurately, but are inaccurate in the prediction of X4-using viruses (~50–70%)^26^. Thus, more accurate tropism prediction methods are required. Here, we present two methods, XGBpred and HMMpred. We analyzed the HIV-1 tropism prediction ability of our methods and compared them with the Geno2pheno, G2p_str, Hivcopred, CM and WebPSSM methods. The results show that XGBpred is robust with the hard-to-predict dual tropic sequences.

## Methods

### Datasets

To construct the Newdb dataset, we extracted 6790 R5 tropic, 590 X4 tropic and 1125 dual tropic sequences from the Los Alamos HIV sequence database (http://www.hiv.lanl.gov/, last update: 10 Sep 2017). The tropisms of the sequences from the Los Alamos HIV sequence database have been phenotypically determined, none of them have been inferred from sequences. Then we removed sequences containing non-canonical residues, reserved sequences with lengths between 31 and 39, and dislodged duplicated sequences to guarantee the high quality of genotype/phenotype pairs. This process finally generated 2335 R5 and 663 X4-using (245 X4 and 418 dual) tropic sequences. The distribution of the six major subtypes in the Newdb dataset is shown in Table 1. To compare our methods with the Geno2pheno, G2p_str, Hivcopred, CM and WebPSSM methods, we used the datasets constructed in these studies, respectively. These datasets can be accessed in Supplementary Spreadsheet S1. The distributions of tropisms in different datasets are shown in Table 2.

**Table 1:** Distribution of the six major subtypes in the Newdb dataset.

Subtype	Number (R^a^, X^b^, D^c^)	Percentage
B	1503 (1209, 93, 201)	50.13%
C	511 (460, 26, 25)	17.04%
D	233 (120, 52, 61)	7.77%
01_AE	213 (149, 45, 19)	7.10%
A	155 (140, 5, 10)	5.17%
02_AG	124 (50, 3, 71)	4.14%

Notes: ^a^The number of R5 tropic sequences. ^b^The number of X4 tropic sequences. ^c^The number of dual tropic sequences.

**Table 2:** Distribution of tropisms in the different datasets.

Dataset	R5	X4-using		Sum
		X4	Dual	
Newdb	2335	245	418	2998
G2p_str^23^	973	94	121	1188
Hivcopred^a^ ^24^	1768	246	321	2335
CM^22^	2354	277	48	2679
WebPSSM^21^	228^b^ (47^c^)	51^b^ (24^c^)		279^b^ (71^c^)

Notes: ^a^Removed 31 duplicated sequences from the original Hivcopred dataset which are marked as not only R5 tropism but also X4-using tropism. ^b^Training set. ^c^Validation set.

### Machine learning method: XGBpred

Extreme gradient boosting (XGboost), like RFs used by T-CUP2^25^, is an ensemble algorithm of decision trees^27^. The ensemble works by combining a set of weaker machine learning algorithms to get an improved machine learning algorithm in overall. The main difference between XGboost and RFs is the way of sampling. RFs are based on uniform sampling with return. Instead, XGboost gives higher weights to the wrongly predicted samples in the current weaker leaner, and then these samples will be paid more attention when training the next weaker leaner. In addition, XGboost adds regularization to avoid overfitting. Therefore, XGboost is a more complicated algorithm than RFs, and thus always outperforms.

Because XGboost is designed for vectors, it is necessary to convert V3 loop string sequences of different lengths to numerical vectors. For this task, we used many kinds of features to describe the characteristics of protein sequences, such as split amino acid composition^24^, dipeptide composition^28^, and net charge or hydropath^29^. We also proposed an additional set of features: the alignment score. The 35-dimensional alignment scores were generated by scoring alignments using the block substitution matrices BLOSUM62, BLOSUM90 or BLOSUM100^30^, and the alignments were generated by aligning sequences to the consensus sequence with 35 residues by the means of Needleman-Wunsch (Version: EMBOSS: 6.6.0)^31^. For the XGBpred method, we tested these different features and their combinations to find the optimal model to discriminate R5 and X4-using sequences.

### Statistics method: HMMpred

Hidden Markov model (HMM) is a finite model applied in time series and linear sequences. Just as the PSSM profile, HMM also can be used to describe protein families. The HMM profile described by state-transition and symbol-emission probabilities performs better than PSSM in terms of sequence alignment and homology recognition because it can deal with gaps in protein families better by hidden state chains^32^.

#### HMM profile construction

We used the maximum likelihood estimation method to establish R5 and X4-using specific HMM profiles from R5 and X4-using tropic multiple sequence alignments generated by ClustalO^33^, respectively. In addition, we simply assigned columns that had more than half gap characters as insertion states. The structure of HMM that we used was no transition allowed from *D*_*j*_ to *I*_*j*_ or from *I*_*j*_ to *D*_*j*+1_ (This kind of structure performed better than the full structure, as shown in Supplementary Table S1). M, D, and I denote match, deletion and insertion states, respectively.

1 $${\hat{a}}_{kl}=\frac{{A}_{kl}+1}{{\sum }_{l^{\prime} }{A}_{kl^{\prime} }+3}$$

2 $${\hat{e}}_{k}(a)=\frac{{E}_{k}(a)+1}{{\sum }_{a^{\prime} }{E}_{k}(a^{\prime} )+21}$$

Where in, k and l are indices over states M, D, or I; a is an amino acid symbol or gap; ${\hat{a}}_{kl}$ means the estimated probability of transiting from state k to state l, ${\hat{e}}_{k}(a)$ means the estimated probability of emitting residue a at state k, and *A*_*kl*_ and *E*_*k*_(*a*) are the corresponding frequencies. In order to avoid the zero probability which represents it cannot happen in the future, we applied the Laplace’s pseudo-count rule that added one to each frequency.

#### Sequence-profile alignment

We employed Viterbi algorithm^34^, a dynamic programing algorithm, to get two alignment scores *S*_*R*5_ and *S*_*non-R*5_. Those alignment scores represent the optimal state pathway scores from the R5 and X4-using HMM profiles, respectively. the final score was defined as:

3 $${\rm{S}}={S}_{R5}-{S}_{non-R5}$$

Then the given sequence would be classified as R5 tropic if the final score S is higher than a threshold, otherwise it would be classified as X4-using tropic.

### Ten-fold cross validation

The widely-used 10-fold cross validation was used to evaluate the performance of our methods in this study, where the sequences were divided into 10 subsets randomly, one subset was used as the testing set, and the others were used as the training set. After ten repetitions, the final performance was average of the performances of those ten subsets.

### Evaluation parameters

For evaluation, we used sensitivity, specificity, accuracy and Matthew’s correlation coefficient (MCC). In particular, MCC is robust even when the size of classes varies widely^35^. An MCC value ‘0’ corresponds to a completely random prediction, while ‘1’ corresponds to a perfect perdition. These parameters were calculated using the following equations:

4 $${\rm{Sensitivity}}=\frac{{\rm{TP}}}{TP+FN}$$

5 $${\rm{Specificity}}=\frac{{\rm{TN}}}{FP+TN}$$

6 $${\rm{Accuracy}}=\frac{{\rm{TP}}+{\rm{TN}}}{TP+FP+TN+FN}$$

7 $${\rm{MCC}}=\frac{{\rm{TP}}\times {\rm{TN}}-{\rm{FP}}\times {\rm{FN}}}{\sqrt{(TP+FP)(TP+FN)(TN+FP)(TN+FN)}}$$

where TP is the number of true positives, FP false positives, TN true negatives and FN false negatives. We regarded R5 tropic samples as positives in this study.

In contrast to the four threshold-dependent parameters, the receiver operating characteristic (ROC) curve, a threshold-independent parameter, illustrates the trade-off between sensitivity and specificity at various threshold settings. In this study, we used the area under the curve (AUC) to measure a predictive power, where 0.5 means a random method, and 1 means a perfect method^36^.

## Results

### Performance on the Newdb dataset

**Figure 1:**
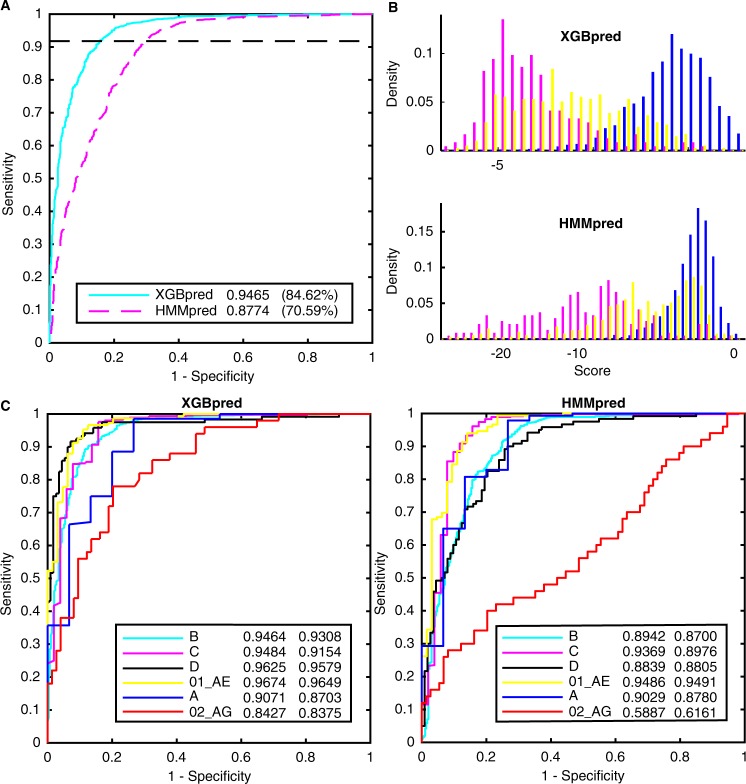
Performance of the XGBpred and HMMpred methods on the Newdb dataset. (**A**) ROC curves on the Newdb dataset in a same 10-fold cross validation test. The legend lists AUCs and specificities at the sensitivity of 91.78% which is plotted as the dashed black line. (**B**) Distribution of V3 loop sequence scores calculated from XGBpred and HMMpred on the Newdb dataset. The score distribution of the R5 tropic sequences is shown in blue, that of X4 is carmine and that of dual is yellow. (**C**) ROC curves of XGBpred and HMMpred for the six major subtypes. The legend lists AUCs and mAPs.

The feature set and the model that gave the strongest predictive power for the XGBpred and HMMpred methods were found, respectively (Supplementary Tables S1 and S2). The performances of the two methods on the Newdb dataset in a same 10-fold cross validation test are shown in Fig. 1A and Table 3. XGBpred had a higher specificity, accuracy, MCC and AUC than HMMpred when having the same sensitivity. Furthermore, the specificity of XGBpred was higher than 80% (84.62%) at the sensitivity of 91.78%. Results from the two methods were highly consistent: they predicted same tropisms for 87.96% of total samples, and achieved 96.70% sensitivity, 83.39% specificity and 93.93% accuracy.

**Table 3:** Performance of the XGBpred and HMMpred methods on the different datasets.

Dataset	Method	Specificity	Accuracy	MCC	AUC
Newdb	XGBpred	84.62%	90.19%	0.7310	0.9465
	HMMpred	70.59%	87.09%	0.6247	0.8774
G2p_str^23^	Geno2pheno^23^	61.6%	—	—	0.860
	G2p_str^23^	68.6%	—	—	0.892
	XGBpred	72.56%	89.90%	0.6605	0.8952
	HMMpred	72.09%	89.81%	0.6570	0.9002
Hivcopred^24^	Hivcopred^24^	81.44%	87.07%	0.67	0.904
	XGBpred	87.13%	88.52%	0.7154	0.9483
	HMMpred	71.08%	84.63%	0.5899	0.8829
CM^22^	CM^22^	92.92%	95.21%	0.885	0.97
	XGBpred	93.85%	95.33%	0.8106	0.9809
	HMMpred	89.54%	94.81%	0.7826	0.9635
WebPSSM^21^	WebPSSM^21^	83.3%	—	—	0.881
	XGBpred	83.33%	83.10%	0.6419	0.9043
	HMMpred	75.00%	80.28%	0.5693	0.8678

Performance of XGBpred and HMMpred on the Newdb, G2p_str, Hivcopred, CM and WebPSSM datasets at the sensitivities of 91.78%, 93.73%, 89.99%, 95.54% and 82.98%, respectively.

Considering the poorer performance of HMMpred, the score distributions of the two methods were plotted (Fig. 1B). As depicted, the scores of dual tropic sequences mostly placed in the middle of the scores of X4 and R5 tropic sequences. Furthermore, HMMpred generated higher scores for a considerable number of dual tropic samples than XGBpred. This phenomenon illustrates that it is hard for dual tropic sequences to be correctly classified, especially by HMMpred.

The performances of the two methods for the six major subtypes (subtypes B, C, D, 01_AE, A and 02_AG) in the Newdb dataset were analyzed due to the sequence divergence among different subtypes and the different number of sequences in each subtype (Fig. 1C). HMMpred for subtypes B and D showed much lower AUCs (0.8942 and 0.8839) than for subtypes C and 01_AE (0.9369 and 0.9486). The reason was that subtypes B and D contained more hard-to-predict dual tropic sequences (Table 1). This also resulted in a low AUC (0.5887) for subtype 02_AG, and a higher AUC (0.9029) for subtype A than for subtype D (0.8839) generated by HMMpred. In contrast, the performance of XGBpred was not so deeply influenced by dual tropic sequences. XGBpred had higher AUCs for the top four most common subtypes (subtypes B, C, D and 01_AE) than for subtypes A and 02_AG. In addition, The V3 loops of subtypes 01_AE and 02_AG come from subtypes E and A, respectively^37,38^. This can also further lead to the weaker predictive power for subtypes A and 02_AG as it is a trickier task to determine tropism for subtype A than the other subtypes^39,40^. Besides, the large biases existed between the number of R5 and X4-using samples for subtypes C and A (Table 1). Therefore, we also reported the mean average precision (mAP) of the two classes (Fig. 1C). AP is the areas under the precision-recall curve for a certain class. The bigger the mAP is, the better the method preforms. The mAPs and AUCs demonstrated the same tendency for the predictive power of our methods. Among all subtypes, just as AUCs, XGBpred and HMMpred showed the highest mAPs (0.9646, 0.9491) for subtype 01_AE. Moreover, for both XGBpred and HMMpred, the divergences between AUCs and mAPs for subtypes C and A were biggest. This may arise from the large biases between the amount of R5 and X4-using samples.

### Comparison with other methods

In this section, to evaluate our methods, we compared with the previously published methods Geno2pheno, G2p_str^23^, Hivcopred^24^, CM^22^, and WebPSSM^21^ by implementing our methods in a 10-fold cross validation test on the datasets used in these published methods, respectively. The exception was WebPSSM^21^ where we used the training set from WebPSSM to model our methods in a 10-fold cross validation test and used the validation set from WebPSSM to test (Table 3).

First when comparing with the Geno2pheno and G2p_str methods^23^, XGBpred and HMMpred achieved AUCs of 0.8952 and 0.9002, respectively. Our methods had higher AUCs than Geno2pheno (0.860) and G2p_str (0.892). In addition, XGBpred and HMMpred achieved specificities of 72.56% and 72.09% at the sensitivity of 93.73%. The specificities were obviously higher than the specificities of Geno2pheno (61.6%) and G2p_str (68.6%) at the same sensitivity. Second, when comparing with the Hivcopred method^24^, XGBpred had a higher AUC (0.9483) than Hivcopred (0.904), but HMMpred had a low AUC (0.8829) as on the Newdb dataset. Third, when comparing with the CM method^22^. Our methods were as accurate as the CM method on the CM dataset which only contains a small amount of hard-to-predict dual tropic samples (Table 2). Finally, when comparing with the WebPSSM method^21^, although the WebPSSM dataset is small, XGBpred had a higher AUC (0.9043) than WebPSSM (0.881), and HMMpred presented a similar AUC (0.8678) with WebPSSM.

### Feature importance analysis

**Figure 2:**
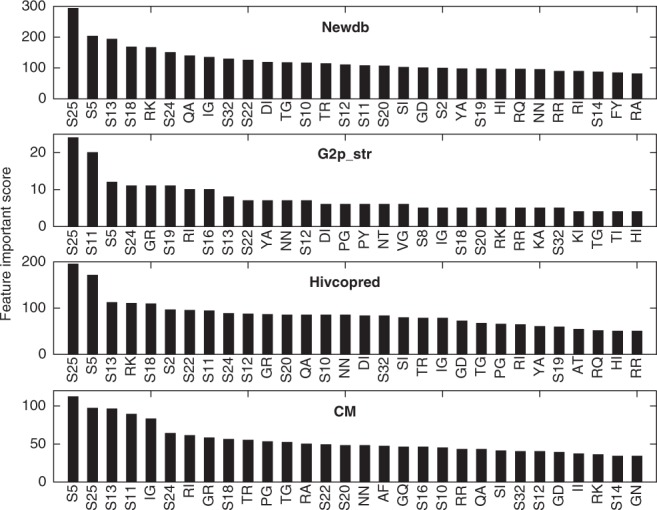
Distribution of feature importance scores. The top 30 most important features indicated by XGBpred on the Newdb, G2p_str, Hivcopred and CM datasets. S# means the alignment score in position #, and R1R2 represents a dipeptide.

Given the high performance of XGBpred presented in the previous subsections, we discussed which features XGBpred provided with its predictive power (Fig. 2). We did not analyze the feature importance on the WebPSSM dataset as it contains few training samples (Table 2). In the XGBpred method, the feature alignment score in the 5^th^ position of the V3 loop appeared in the top three most important features on all datasets. Interestingly, amino acid Tyr in position 5 appeared more frequently in X4-using tropic than in R5 tropic sequences (Supplementary Fig. S1). Currently, X4-using tropism can be predicted by the 11/25 rule^19^. However, since position 5 was as same important as positions 11 and 25, the pragmatic 11/25/5 rule was proposed to predict a virus as X4-using tropic by the presence of a positively charged amino acid in positions 11 or 25, or by the presence of amino acid Tyr in position 5 of its V3 loop. Compared with the 11/25 rule, the 11/25/5 rule reduced sensitivities by 1.29%, 1.03%, 1.14% and 1.19% on the Newdb, G2p_str, Hivcopred and CM datasets while increasing specificities by 7.39%, 5.11%, 6.34% and 10.77%, respectively. The 11/25/5 rule also had higher accuracies and MCCs than the 11/25 rule on the four datasets, which indicates the influence of amino acid Tyr in position 5 with regard to viral tropism (Supplementary Table S3). In addition to positions 5, 11 and 25, positions 13, 18, 22 and 24 also ranked in the top ten most important features on the four datasets. Two exceptions were position 18 ranked 21^st^ on the G2p_str dataset, and position 22 ranked 14^th^ on the CM dataset. Indeed, all the positions that we identified as correlated with HIV-1 tropism are exactly in accordance with the results from Sander *et al*.^41^ who point to the residues 298 (3), 302 (7), 306 (11), 308 (13), 315 (18), 317 (20), 319 (22), 321 (24), 322 (25) and 328 (32) are important for tropism. Furthermore, the feature importance distribution generated by XGBpred is a feasible method to judge whether a new discovered association pattern is of importance to co-receptor usage or not.

## Discussion

In this study, we present two methods, XGBpred and HMMpred, for HIV-1 co-receptor usage prediction. XGBpred is based on machine learning, and HMMpred is based on statistics. XGBpred performed best on the Hivcopred and Newdb datasets containing larger proportions of hard-to-predict dual tropic samples in the X4-using samples, while HMMpred performed worst. In contrast, the predictive powers of the two methods were similar on the smaller G2p_str and CM datasets containing fewer dual tropic samples (Table 3). The poor ability of HMMpred to predict tropism stemmed from the high probability that HMMpred incorrectly predicted dual tropic samples as R5 tropic (Fig. 1B and Supplementary Fig. S2). The profiles used in HMMpred may not be meticulous enough. Several reasons may account for this phenomenon. Firstly, the two sequence families are highly similar since even one amino acid substitution may change their tropisms^42,43^. Secondly, the characteristics of dual tropic sequences may be overwhelmed by R5 and X4 tropic sequences. Finally, the unavailability of X4-using tropic samples makes it uncertain to learn its accurate HMM profile. Moreover, as the number of samples increased, the gap of predictive powers between XGBpred and HMMpred became large (Tables 2 and 3). This corresponds to the fact that the machine learning based Geno2pheno method is more widely used than the statistics based 11/25 rule and WebPSSM. As a result, a machine learning based method, in particular XGBpred, is recommended to predict co-receptor usage as the number of samples continues to expand.

In an effort to further increase the predictive power, we also generated three meta methods by the means of stacking^44^. The scores generated by XGBpred, Hivcopred (SVM^light^) and HMMpred were added as additional features to the new stacking based XGBpred models. Compared with the original XGBpred method, the new stacking-based XGBpred methods had slightly higher AUCs on the G2p_str dataset but lower AUCs on the other datasets (Supplementary Table S4). The poor performances of the meta methods may due to the poorer predictive abilities of Hivcopred and HMMpred than the original XGBpred method, and/or the dependence of the results generated by XGBpred, Hivcopred and HMMpred (Supplementary Table S5). This may stem from the fact that the V3 loop is not the sole determinant of viral tropism. Moreover, V1, V2, C4 and the bridge sheet regions of gp120 also have an impact on co-receptor usage^45,46^. To predict tropism, several methods gain a higher accuracy by employing other information in addition to the V3 loop, such as clinical information^47^, V2 loop sequences^48^ and structure information^23,25,41^. Therefore, the stacking based method can be constructed to improve its predictive power by combining methods with different kinds of information.

In summary, the two methods we developed performed comparably on the datasets containing less hard-to-predict dual tropic sequences, but XGBpred performed much better on the datasets with more dual tropic sequences. This means XGBpred is more robust to predict dual tropic sequences than other methods. Thus, we strongly recommend to use XGBpred to predict viral tropism. Our two methods have been implemented as a freely available webserver under http://spg.med.tsinghua.edu.cn:23334/XGBpred/.

## Supplementary information

Figures S1, S2; Tables S1, S2, S3, S4, S5

Supplementary Spreadsheet S1
