# Supplementary material for: HIV-1 tropism prediction by the XGboost and HMM methods
Source: Sci Rep. 2019 Jul 10;9:9997. doi: 10.1038/s41598-019-46420-4 (PMC6620319; doi:10.1038/s41598-019-46420-4)
Supplement: Supplementary file 1 — Figures S1, S2; Tables S1, S2, S3, S4, S5 [file 41598_2019_46420_MOESM1_ESM.pdf]

HIV-1 tropism prediction by the XGboost and HMM methods

Xiang Chen[1], Zhi-Xin Wang[1], Xian-Ming Pan[*]

[1]Key Laboratory of Ministry of Education for Protein Science, School of Life Sciences, Tsinghua University, Beijing 100084, China.

[*]Correspondence to Xian-Ming Pan, Key Laboratory of Bioinformatics, Ministry of Education, School of Life Sciences, Tsinghua University, Beijing 100084, China.

Tel: +86-10-62792827; Fax: +86-10-62792827

E-mail: pan-xm@mail.tsinghua.edu.cn

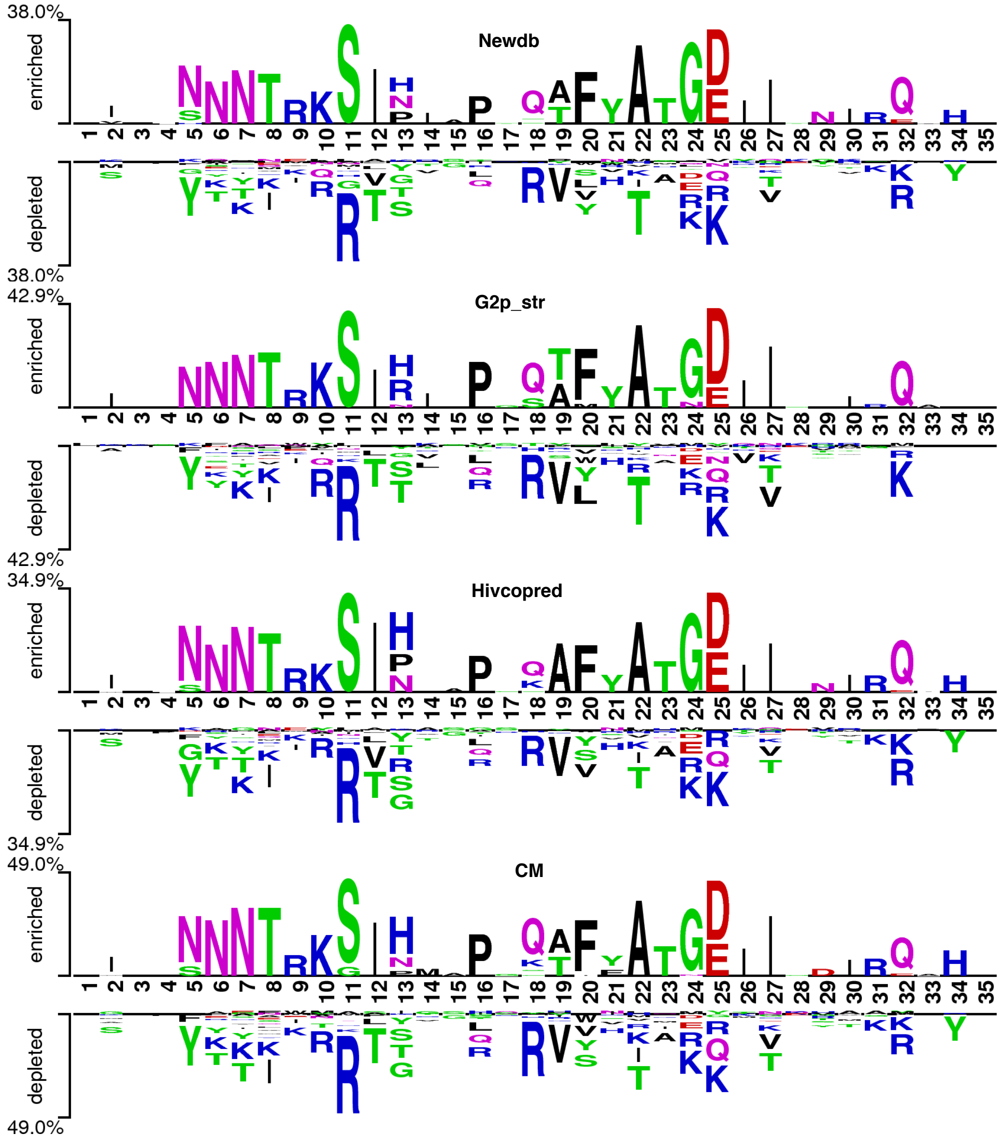

Figure S1. Two sample logos for R5 and X4-using tropic sequences.

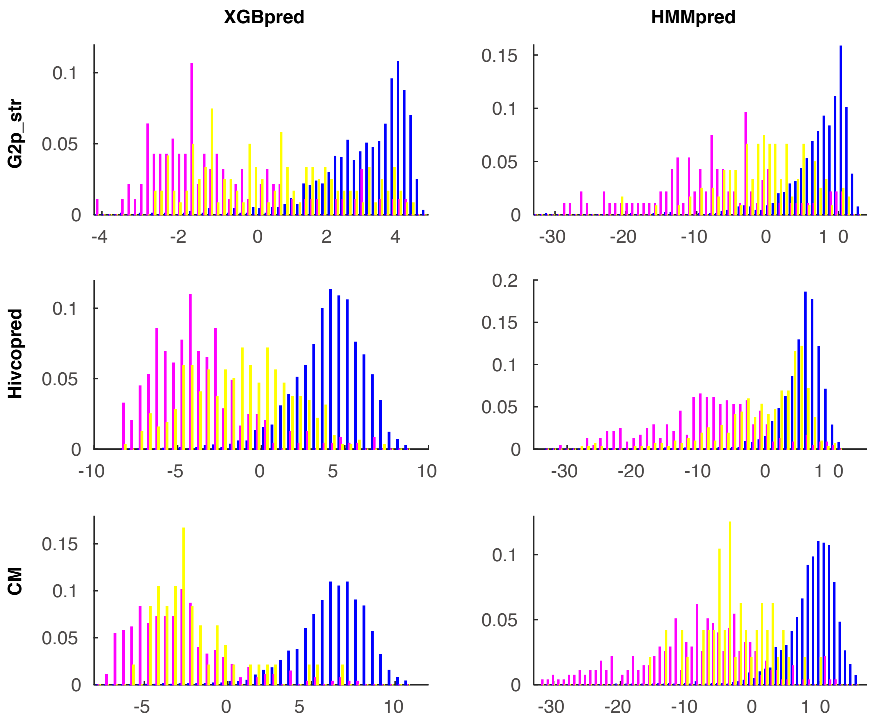

Figure S2. Distribution of V3 loop sequence scores calculated from the XGBpred and HMMpred methods on the G2p_str, Hivcopred and CM datasets. The score distribution of the R5 tropic sequences is shown in blue, that of X4 is carmine and that of dual is yellow.

Table S1. Construction of the HMMpred method. Performances of the HMMpred method based on different HMM models on the Newdb dataset in a same 10-fold cross validation test (threshold = 1.0). Notes: [a]The full HMM model: transition allowed from D_j to I_j or from I_j to D_{j+1}. [b]The MSA generated by EMBOSS was manually adjusted to 35 match states. [c]No emission allowed in insertion states. [d]The HMMER model: no transition allowed from D_j to I_j or from I_j to D_{j+1}. [e]Regardless of background frequencies. [*]The final HMM model used in this study.

Model	Sensitivity	Specificity	Accuracy	MCC	AUC
**Full[a]**	92.76%	67.57%	87.19%	0.6194	0.8659
**Full_MSA[ab]**	92.42%	67.87%	86.99%	0.6153	0.8669
**I_emission[abc]**	92.21%	66.21%	86.46%	0.5983	0.8628
**HMMER[bd]**	91.18%	69.38%	86.36%	0.6046	0.8686
**No_back1[abe]**	92.89%	69.08%	87.63%	0.6335	0.8764
**No_back2[bde*]**	92.03%	70.29%	87.22%	0.6270	0.8774

Table S2. Construction of the XGBpred method. Performances of the XGBpred method based on different and combination of feature sets on the Newdb dataset in a same 10-fold cross validation test at the sensitivity of 91.78%. Notes: [a]20-dimensional amino acid composition feature set. [b]Split amino acid composition feature sets: [b1]40-d split amino acid composition; and [b2]combining with 1-d full net charge; and [b3]combing with 6-d full and split net charges and hydropath; [b4]60-d split amino acid composition. [c]35-d alignment score feature sets: using blocks substitution matrix [c1]BLOSUM62, [c2]BLOSUM90 or [c3]BLOSUM100, respectively. [bc]Combinational feature sets of 40-d

split amino acid composition and 35-d alignment score features. [ac]Combinational feature sets of 20-d amino acid composition and 35-d alignment score features. [d]400-d dipeptide composition feature set. [dc]Combinational feature sets of 400-d dipeptide composition and 35-d alignment score features. [*]The final feature set used in the XGBpred method.

Features	Specificity	Accuracy	MCC	AUC
**20d[a]**	76.02%	88.29%	0.6664	0.9151
**40d[b1]**	80.84%	89.36%	0.7029	0.9256
**40d1d[b1]**	82.50%	89.69%	0.7146	0.9267
**40d2d[b2]**	82.35%	89.69%	0.7142	0.9250
**40d6d[b3]**	79.79%	89.13%	0.6950	0.9180
**60d[b4]**	82.20%	89.66%	0.7131	0.9324
**35d (B62)[c1]**	83.56%	89.96%	0.7232	0.9324
**35d (B90)[c2]**	82.35%	89.69%	0.7142	0.9339
**35d (B100)[c3]**	84.01%	90.06%	0.7266	0.9386
**40d35d (B100)[bc]**	84.62%	90.19%	0.7310	0.9413
**40d35d (B90)[bc]**	83.56%	89.96%	0.7232	0.9371
**20d35d (B100)[ac]**	83.86%	90.03%	0.7254	0.9370
**400d[d]**	82.96%	89.83%	0.7187	0.9432
**400d35d (B100)[dc*]**	**84.62%**	**90.19%**	**0.7310**	**0.9465**

Table S3. Performance of the 11/25 and 11/25/5 rules on different datasets.

Dataset	Rule	Sensitivity	Specificity	Accuracy	MCC
**Newdb**	11/25	94.90%	50.53%	85.09%	0.5260
	11/25/5	93.79%	57.92%	85.86%	0.5630
**G2p_str**	11/25	94.86%	54.42%	87.54%	0.5459
	11/25/5	93.83%	59.53%	87.63%	0.5630
**Hivcopred**	11/25	95.26%	47.80%	83.81%	0.5166
	11/25/5	94.40%	54.14%	84.63%	0.5492
**cm**	11/25	95.03%	61.23%	90.93%	0.5695
	11/25/5	93.84%	72.00%	91.19%	0.6168

Table S4. Performance of stacking based meta methods. Performance of stacking based XGBpred methods in a same 10-fold cross validation test at the sensitivity of 91.78%, 93.73%, 88.97% and 95.54% on the Newdb, G2p_str, Hivcopred and CM datasets, respectively. Notes: [a]3-d XGBpred, Hivcopred and HMMpred score feature set. [b]Combinational feature sets of 400-d dipeptide composition, 35-d alignment score features, and 2-d Hivcopred and HMMpred score features. [c]Combinational feature sets of 400-d dipeptide composition, 35-d alignment score features, and 1-d Hivcopred score features (considering the poor performance of HMMpred).

Dataset	Method	Specificity	Accuracy	MCC	AUC
**Newdb**	3d[a]	84.62%	90.19%	0.7310	0.9453
	435d2d[b]	84.31%	90.13%	0.7288	0.9362
	435d1d[c]	83.56%	89.96%	0.7232	0.9326
**G2p_str**	3d	73.49%	90.07%	0.6674	0.8978
	435d2d	73.02%	89.98%	0.6640	0.8993
	435d1d	72.09%	89.81%	0.6570	0.9040
**Hivcopred**	3d	89.07%	88.99%	0.7303	0.9467
	435d2d	85.54%	88.14%	0.7032	0.9409
	435d1d	86.60%	88.39%	0.7114	0.9392
**CM**	3d	95.08%	95.48%	0.8185	0.9778
	435d2d	94.77%	95.45%	0.8165	0.9782
	435d1d	94.15%	95.37%	0.8126	0.9797

Table S5. Dependence of results generated by XGBpred, Hivcopred and HMMpred. Pearson correlation analysis and statistical hypothesis test (two-tailed t-test by SPSS) for samples predicted wrongly by the XGBpred method.

Dataset	Methods	XGBpred	
		Pearson correlation	P value
**Newdb**	Hivcopred	0.578	<0.01
	HMMpred	0.506	<0.01
**G2p_str**	Hivcopred	0.657	<0.01
	HMMpred	0.741	<0.01
**Hivcopred**	Hivcopred	0.578	<0.01
	HMMpred	0.488	<0.01
**CM**	Hivcopred	0.642	<0.01
	HMMpred	0.516	<0.01
